# Epigenetic remodeling in insect immune memory

**DOI:** 10.3389/fimmu.2024.1397521

**Published:** 2024-06-10

**Authors:** Krishnendu Mukherjee, Ulrich Dobrindt

**Affiliations:** Institute of Hygiene, University of Münster, Münster, Germany

**Keywords:** epigenetics, DNA methylation, histone acetylation, immune memory, immune priming, insect resistance

## Abstract

The innate immune system of insects can respond more swiftly and efficiently to pathogens based on previous experience of encountering antigens. The understanding of molecular mechanisms governing immune priming, a form of immune memory in insects, including its transgenerational inheritance, remains elusive. It is still unclear if the enhanced expression of immune genes observed in primed insects can persist and be regulated through changes in chromatin structure via epigenetic modifications of DNA or histones, mirroring observations in mammals. Increasing experimental evidence suggests that epigenetic changes at the level of DNA/RNA methylation and histone acetylation can modulate the activation of insects’ immune responses to pathogen exposure. Moreover, transgenerational inheritance of certain epigenetic modifications in model insect hosts can influence the transmission of pre-programmed immune responses to the offspring, leading to the development of evolved resistance. Epigenetic research in model insect hosts is on the brink of significant progress in the mechanistic understanding of chromatin remodeling within innate immunity, particularly the direct relationships between immunological priming and epigenetic alterations. In this review, we discuss the latest discoveries concerning the involvement of DNA methylation and histone acetylation in shaping the development, maintenance, and inheritance of immune memory in insects, culminating in the evolution of resistance against pathogens.

## Introduction

Immunological memory is characterized by the adaptive capacity of the immune system to respond more swiftly and efficiently to previously encountered pathogens. The conventional notion that immunological memory is solely attributed to the adaptive immune system of mammals is being questioned by growing evidence on diverse forms of immune memory across various organisms, including invertebrate species (1, 2). For example, the long-standing notion that insects only induce their basal immune function during infection has been replaced by evidence showing that the innate immune system in some insect species can generate memory responses to subsequent reinfections by the same or different pathogens, despite potential fitness costs (3–5). The efficiency of innate immune clearance can be modulated by previous encounters with microbes or microbial products, leading to an elevation in the immune response, rendering the insect resistant to a subsequent lethal infection a short time later. This phenomenon, known as ‘immune priming’ parallels the memory and specificity seen in the trained immunity of vertebrates (1, 5). Insects can improve the survival of offspring exposed to the same pathogens their parents encountered by transgenerational immune priming (TGIP). However, immune priming is not seen in all insect species, and there is less experimental evidence for TGIP compared to within-generational priming in insects (3–5). Nevertheless, most of the research on immune priming tends to emphasize alterations in survival or reproduction rates following repeated pathogen exposure, without offering a comprehensive understanding of the involved molecular mechanisms.

The distinctive and targeted nature of immune priming has led to the investigation of specific genes as a potential catalyst for bolstering immunity. The pattern recognition receptors (PRRs), antimicrobial peptides (AMPs), and Down syndrome cell adhesion molecule (Dscam) are most investigated as core components of the immune priming molecular network (4). Beyond gene-specific investigations, immune priming reveals a unique pattern of enhanced or suppressed gene expression, which significantly diverges from the pattern seen after an infection in the absence of priming (6). The molecular underpinnings of the modified responsiveness observed in a specific subset of innate immunity genes remain only partially comprehended. Nevertheless, accumulating evidence indicates the convergence of multiple regulatory layers in this phenomenon, comprising alterations in chromatin organization (7). Epigenetic mechanisms like DNA/RNA methylation and histone acetylation encompass a set of biochemical modifications that alter the individual phenotype by inducing alterations in gene expression without changing the underlying DNA sequence (8). DNA methylation and histone acetylation exhibit conservation across both mammals and insects, instigating alterations in chromatin structure by disrupting the electrostatic interactions between cationic histone tails and the anionic DNA phosphodiester backbone. Here, we discuss the available limited number of research articles of importance that have identified the significance of epigenetic reprogramming at the level of DNA methylation and histone acetylation within subsequent generations of immune-primed insects.

## Regulation of DNA methylation and histone acetylation during activation of insect immunity

Epigenetic modifications linked to the initial antimicrobial response in insects upon exposure to dead or sublethal levels of microbes serve as the foundational mechanism for maintaining cell identity and establishing long-term cellular memory (9). DNA methylation involves adding methyl groups to cytidine residues, regulating gene expression (10). This process is maintained by DNA methyltransferases (DNMTs), with DNMT1 preserving methylation patterns and DNMT3 establishing new marks (10). DNMT2 methylates transfer RNA (tRNA) instead of DNA (10). Besides, methylated RNA includes m6A, m5C, m7G, and 2-O-methylation, but their specific biological significance to insect immunity is yet to be established (11). Infection with the entomopathogenic bacterium *Bacillus thuringiensis* (Bt) stimulates the expression of DNMT genes as well as antimicrobial genes in the larvae of the cotton bollworm *Helicoverpa armigera* (12). Downregulation of DNMT genes is associated with suppression of antimicrobial gene expression, rendering the greater wax moth *Galleria mellonella* vulnerable to parasitism (13). DNA methylation levels may influence the different susceptibility of two naturally occurring phenotypes of the mosquito *Anopheles albimanus* to *Plasmodium berghei* (14).

Increased immune gene expression in insects during initial pathogen encounter is also associated with changes in histone acetylation, which is qualitatively (type of histones) and quantitatively (acetylation levels) different from their uninfected counterparts (15, 16). Modulating the positive charge density of core histones, primarily through acetylation, regulates the accessibility of DNA, which is essential for transcriptional activity. Acetylated histones promote a loose, accessible chromatin structure conducive to gene expression, while deacetylated histones lead to tighter DNA binding and compaction, rendering it transcriptionally silent. Histone acetylation and deacetylation are governed by opposing actions of histone acetyltransferases (HATs) and histone deacetylases (HDACs). The entomopathogenic fungus *Metarhizium robertsii* and the human bacterial pathogen *Listeria monocytogenes* can regulate innate immune response in *G. mellonella* by altering the balance of HDAC-HAT activity (15). HDACs exhibited a greater induction compared to HATs during infection, and the HDAC-HAT imbalance endured notably longer in larvae infected with pathogenic bacteria compared to non-pathogenic *E. coli* (15, 16). These findings suggest that epigenetic changes may regulate the initiation of the initial immune response in insects and could directly influence immune priming against similar or distinct pathogens.

## DNA methylation and histone acetylation-mediated regulation of immune priming

### Immune priming within a single generation

**Figure 1:**
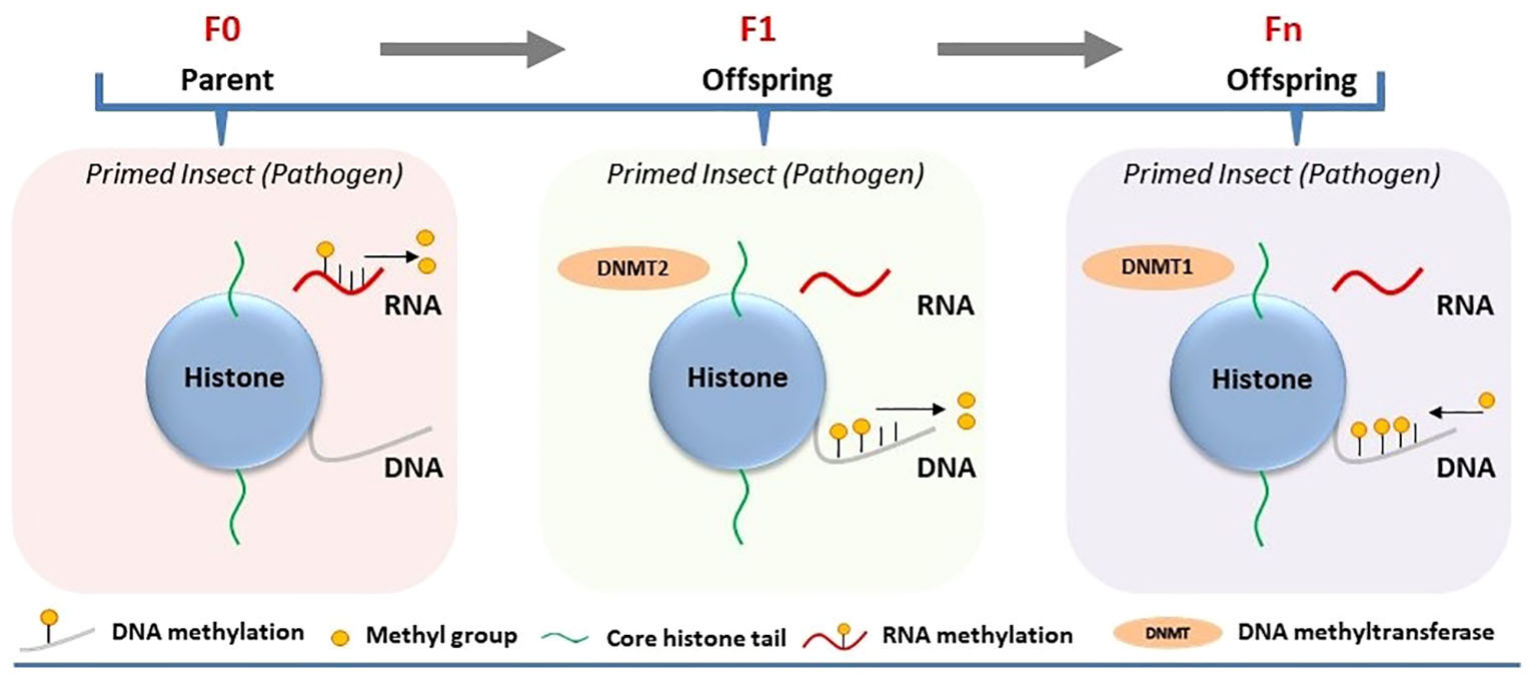
Involvement of DNA/RNA methylation in the regulation of immune priming in insects. Immune priming against pathogens in the parental generation (F0) leads to decreased RNA methylation, while parental transmission of immune priming to their offspring (intergenerational or F1) results in reduced DNA methylation and increased *Dnmt2* expression, with subsequent generations (Fn) showing elevated DNA methylation.

Experimental demonstrations of epigenetic regulations tied to immune priming within a single generation are sparse (17). DNA and RNA methylation was analyzed in the immune-primed mealworm beetle *Tenebrio molitor* after a second infection with the bacterium *Micrococcus lysodeikticus*. Immune priming in male and female beetles against *M. lysodeikticus* infection was associated with reduced RNA methylation (5mC) compared to the control (18) (**Figure 1**). Simultaneously, RNA methylation was analyzed in immune-primed larvae of *T. molitor* after exposure to the entomopathogenic fungus *Metarhizium*. Like adult beetles, immune-primed larvae also displayed different RNA methylation levels compared to the control. The degree of RNA methylation was only restricted within a single generation, as experiments designed to measure its intergenerational inheritance showed no significant difference between the priming group and the control group. However, attempts to analyze the regulatory role of DNA methylation within a single generation of immune-primed beetles proved unsuccessful (5, 18). Nevertheless, this does not eliminate the prospect that alternative epigenetic alterations, like m6A or histone acetylation, might also govern immune priming within the same generation, alongside 5mC RNA methylation.

**Figure 2:**
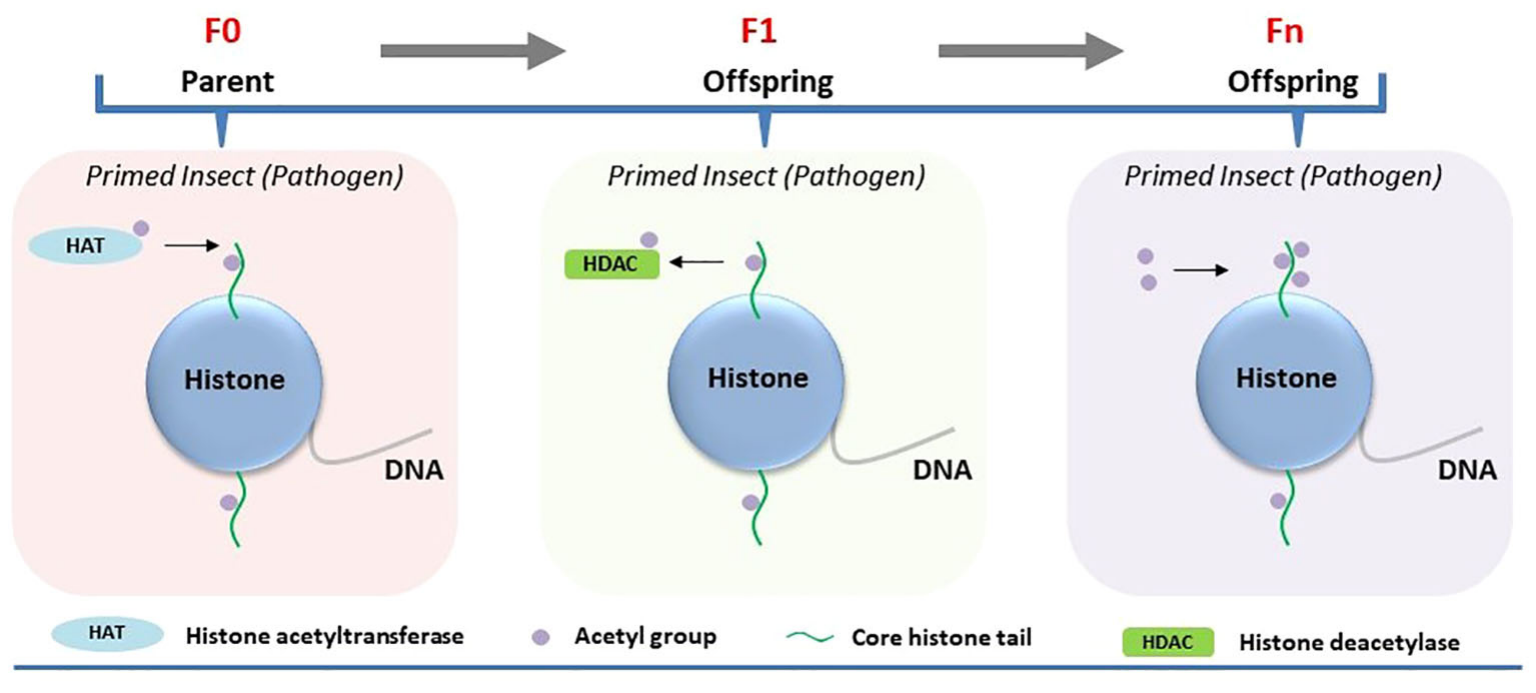
Involvement of histone acetylation in the regulation of immune priming in insects. Immune priming against pathogens in the parental generation (F0) leads to heightened HAT activity. Parental transmission of immune priming against pathogens to their offspring (intergenerational or F1) reduced HDACs. The transmission of immune priming to subsequent generations (Fn) results in elevated H3 acetylation.

Histone acetylation has been shown to sustain enduring alterations in transcriptional regulation in immune-primed insects, such as mosquitoes (19). *Plasmodium* infection induces a long-lasting priming response that enhances antiplasmodial immunity in *Anopheles gambiae*, the primary vector of malaria in Africa (19). *Plasmodium* midgut invasion allows prostaglandin E2 (PGE2)-dependent release of hemocyte differentiation factor (HDF) by midgut epithelial cells to enhance the circulation of granulocytes and oenocytes in immune-primed mosquitoes. A double peroxidase (DBLOX) enzyme is essential for HDF synthesis and DBLOX silencing completely abolished the priming response to infection (19). Besides, immune priming is mediated by HATs, which are known to catalyze the transfer of an acetyl group to histones, promoting gene expression. Different HATs expressed in *A. gambiae* were evaluated for their role in maintaining immune priming following *Plasmodium berghei* infection. Regulation of immune priming by HATs was only evident for the HAT Tip60, as its gene silencing in *A. gambie* abolished immune priming (**Figure 2**). This finding indicates that Tip60 is required for the synthesis of the hemocyte differentiation factor (HDF) that increases oenocyte numbers in immune-primed mosquitoes (19).

### Immune priming at the intergenerational level

Certain epigenetic marks in insects may evade the reprogramming process that usually eliminates all epigenetic data during gametogenesis, enabling these markers to persist through meiosis and be inherited by offspring (20). It raises the possibility that epigenetic changes occurring in immune-primed insects within a generation can be passed on to germ cells and subsequently transmitted to offspring, thereby influencing immune priming of the next generation. Inheritance of epigenetic changes has been experimentally demonstrated to be associated with immune priming at the intergenerational level (parental to offsprings/F1). Feeding *Manduca sexta* with the entomopathogenic bacterium *Serratia entomophila* or non-pathogenic *E. coli* delayed transformation from larvae to pupae (21). Induction of immune response upon exposure to pathogenic and non-pathogenic bacteria in the infected host was associated with shifts in both DNA methylation and histone acetylation. Maternal intergenerational immune priming was mediated by the translocation of bacterial structures from the gut lumen to the eggs, resulting in microbe-specific transcriptional reprogramming of gene expression in larvae of the F1 generation. For example, significant downregulation of HDAC4 and HAT chameau was observed only in the female offspring of parents infected with *S. entomophila* in the F1 generation (21) (**Figure 2**). On the other hand, the third-instar F1 larvae displayed sex-specific differences in the expression profiles of immunity-related genes and DNA methylation. Exposure to *E. coli* in the parental generation induced sex-specific differences in the expression of DNMT1 and DNMT2 in F1 larvae. The DNA methylation level, however, was reduced in the F1 generation male and female offspring of both parents in both treatment groups (**Figure 1**).

DNMTs may also interfere with the paternal transfer of immunity to the F1 offspring. In the red flour beetle *Tribolium castaneum*, *Dnmt2* expression in the testes was higher than in the wholebody of male beetles of the parental generation (22). RNAi knockdown of the *Dnmt2* gene in fathers had systemic consequences, leading to a slowdown in the developmental pace of offspring larvae and an increased mortality rate among adult offspring when exposed to Bt infection. However, *Dnmt2* silencing in the paternal generation did not affect *Dnmt2* expression in the offspring generation. Also, expression of selected innate immunity genes (*hsp83*, *nimB*, and *PGRP*) was unaffected in the paternal and offspring generations due to *Dnmt2* knockdown (22). Despite the limitations in the mechanistic understanding, this finding highlights the involvement of *Dnmt2* in paternal effects, wherein its knockdown increases the susceptibility of beetle offspring to pathogens.

### Immune priming at the transgenerational level

Recently, the persistence of pathogen-induced epigenetic alterations in immune-primed insects beyond a single generation has been investigated in *G. mellonella*. The larvae were consistently exposed to either Bt or *M. robertsii* across multiple generations, resulting in the emergence of a first generation with heightened resistance to infection (resistant line) compared to susceptible larvae not subjected to selection (susceptible line) (23, 24). Notably, these resistant larvae exhibited various intra-species immuno-physiological adaptations, such as alterations in gut microbiota, melanization, circulating hemocytes, cuticle thickness, and differential expression of immune genes compared to the susceptible line. This phenomenon sparked deeper inquiries into the involvement of DNA methylation and histone acetylation in the evolutionary process underlying *G. mellonella*’s resistance to Bt and *M. robertsii* (23, 24). The investigations unveiled distinct alterations in DNA methylation and histone acetylation patterns across tissues of both resistant (selected line) and susceptible (non-selected line) larvae. Particularly noteworthy were tissue-specific changes observed in the midgut, cuticle, and fat body regions. Bt or *M. robertsii*-resistant larvae showcased heightened levels of histone H3 acetylation in specific tissues: the midgut exhibited increased acetylation in the case of resistance to Bt, while the cuticle displayed elevated acetylation levels in response to resistance against *M. robertsii*, as opposed to susceptible larvae (**Figure 2**). Likewise, there was a tissue-specific elevation in 5-methylcytosine levels in uninfected resistant larvae compared to uninfected susceptible larvae (**Figure 1**). Increased expression of selected histone acetylation and DNA methylation related genes were upregulated in uninfected resistant larvae compared to uninfected susceptible larvae. However, the expression levels of these genes were reversed upon infection of the resistant and susceptible lines with either Bt or *M. robertsii* (23, 24).

## Conclusions

Understanding immune priming in insects requires thorough investigation of epigenetic factors, along with other mechanisms like endoreplication (25, 26). Unresolved research questions include the roles of germline and somatic epigenetic changes, cell and tissue-specific alterations, and the influence of genetic variability on the epigenome. Future studies on immune priming should also encompass insect species that do not manifest priming responses within a single generation, as the effects may be more pronounced across generations through epigenetic inheritance. Adopting a multi-omic approach, including technologies like enzymatic methyl-seq and single-cell sequencing, will enhance the identification and validation of epigenetic signatures in immune-primed insects, augmenting our understanding of host gene expression regulation during immune priming and insect resistance evolution.

## Author contributions

KM: Conceptualization, Data curation, Funding acquisition, Writing – original draft, Writing – review & editing. UD: Conceptualization, Data curation, Funding acquisition, Writing – original draft, Writing – review & editing.

## References

[1] Netea MG Joosten LA Latz E Mills KH Natoli G Stunnenberg HG. Trained immunity: A program of innate immune memory in health and disease. Science. (2016) 352:aaf1098. doi: 10.1126/science.aaf1098 27102489 PMC5087274

[2] Netea MG Domínguez-Andrés J Barreiro LB Chavakis T Divangahi M Fuchs E. Defining trained immunity and its role in health and disease. Nat Rev Immunol. (2020) 20:375–88. doi: 10.1038/s41577-020-0285-6 PMC7186935 32132681

[3] Prakash A Khan I. Why do insects evolve immune priming? A search for crossroads. Dev Comp Immunol. (2022) 126:104246. doi: 10.1016/j.dci.2021.104246 34453994 PMC7614680

[4] Sułek M Kordaczuk J Wojda I. Current understanding of immune priming phenomena in insects. J Invertebr Pathol. (2021) 185:107656. doi: 10.1016/j.jip.2021.107656 34464656

[5] Contreras-Garduño J Lanz-Mendoza H Franco B Nava A Pedraza-Reyes M Canales-Lazcano J. Insect immune priming: ecology and experimental evidences. Ecol Entomol. (2016) 41:351–66. doi: 10.1111/een.12300

[6] Greenwood JM Milutinović B Peuß R Behrens S Esser D Rosenstiel P. Oral immune priming with *Bacillus thuringiensis* induces a shift in the gene expression of *Tribolium castaneum* larvae. BMC Genomics. (2017) 18:329. doi: 10.1186/s12864-017-3705-7 28446171 PMC5405463

[7] Vilcinskas A. Mechanisms of transgenerational immune priming in insects. Dev Comp Immunol. (2021) 124:104205. doi: 10.1016/j.dci.2021.104205 34260954

[8] Palli SR. Epigenetic regulation of post-embryonic development. Curr Opin Insect Sci. (2021) 43:63–9. doi: 10.1016/j.cois.2020.09.011 PMC8044252 33068783

[9] Mukherjee K Dobrindt U. The emerging role of epigenetic mechanisms in insect defense against pathogens. Curr Opin Insect Sci. (2022) 49:8–14. doi: 10.1016/j.cois.2021.10.004 34710642

[10] Lyko F. The DNA methyltransferase family: a versatile toolkit for epigenetic regulation. Nat Rev Genet. (2018) 19:81–92. doi: 10.1038/nrg.2017.80 29033456

[11] Zaccara S Ries RJ Jaffrey SR. Reading, writing and erasing mRNA methylation. Nat Rev Mol Cell Biol. (2019) 20:608–24. doi: 10.1038/s41580-019-0168-5 31520073

[12] Baradaran E Moharramipour S Asgari S Mehrabadi M. Induction of DNA methyltransferase genes in *Helicoverpa armigera* following injection of pathogenic bacteria modulates expression of antimicrobial peptides and affects bacterial proliferation. J Insect Physiol. (2019) 118:103939. doi: 10.1016/j.jinsphys.2019.103939 31493391

[13] Özbek R Mukherjee K Uçkan F Vilcinskas A. Reprograming of epigenetic mechanisms controlling host insect immunity and development in response to egg-laying by a parasitoid wasp. Proc Biol Sci. (2020) 287:20200704. doi: 10.1098/rspb.2020.0704 32519598 PMC7341927

[14] Claudio-Piedras F Recio-Tótoro B Condé R Hernández-Tablas JM Hurtado-Sil G Lanz-Mendoza H. DNA Methylation in Anopheles albimanus modulates the midgut immune response against *Plasmodium berghei* . Front Immunol. (2020) 10:3025. doi: 10.3389/fimmu.2019.03025 31993053 PMC6970940

[15] Mukherjee K Fischer R Vilcinskas A. Histone acetylation mediates epigenetic regulation of transcriptional reprogramming in insects during metamorphosis, wounding and infection. Front Zool. (2012) 9:25. doi: 10.1186/1742-9994-9-25 23035888 PMC3538701

[16] Heitmueller M Billion A Dobrindt U Vilcinskas A Mukherjee K. Epigenetic Mechanisms Regulate Innate Immunity against Uropathogenic and commensal-like *Escherichia coli* in the surrogate insect model Galleria mellonella. Infect Immun. (2017) 85:e00336–17. doi: 10.1128/IAI.00336-17 PMC5607417 28739824

[17] Ottaviani E Invertebrate Surviv J. Invertebrate immunological memory: could the epigenetic changes play the part of lymphocytes? Invertebrate Surviv J. (2015) 12:1–4.

[18] Castro-Vargas C Linares-López C López-Torres A Wrobel K Torres-Guzmán JC Hernández GAG. Methylation on RNA: A potential mechanism related to immune priming within but not across generations. Front Microbiol. (2017) 8:473. doi: 10.3389/fmicb.2017.00473 28400750 PMC5368179

[19] Gomes FM Tyner MDW Barletta ABF Saha B Yenkoidiok-Douti L Canepa GE. Double peroxidase and histone acetyltransferase AgTip60 maintain innate immune memory in primed mosquitoes. Proc Natl Acad Sci U.S.A. (2021) 118:e2114242118. doi: 10.1073/pnas.2114242118 34711682 PMC8612215

[20] Zenk F Loeser E Schiavo R Kilpert F Bogdanović O Iovino N. Germ line-inherited H3K27me3 restricts enhancer function during maternal-to-zygotic transition. Science. (2017) 357:212–6. doi: 10.1126/science.aam5339 28706074

[21] Gegner J Baudach A Mukherjee K Halitschke R Vogel H Vilcinskas A. Epigenetic mechanisms are involved in sex-specific trans-generational immune priming in the lepidopteran model host Manduca sexta. Front Physiol. (2019) 10:137. doi: 10.3389/fphys.2019.00137 30886585 PMC6410660

[22] Schulz NKE Mohamed FF Lo LK Peuß R de Buhr MF Kurtz J. Paternal knockdown of tRNA(cytosine-5-)-methyltransferase (*Dnmt2*) increases offspring susceptibility to infection in red flour beetles. Insect Mol Biol. (2022) 31:711–21. doi: 10.1111/imb.12798 35790040

[23] Mukherjee K Dubovskiy I Grizanova E Lehmann R Vilcinskas A. Epigenetic mechanisms mediate the experimental evolution of resistance against parasitic fungi in the greater wax moth Galleria mellonella. Sci Rep. (2019) 9:1626. doi: 10.1038/s41598-018-36829-8 30733453 PMC6367475

[24] Mukherjee K Grizanova E Chertkova E Lehmann R Dubovskiy I Vilcinskas A. Experimental evolution of resistance against *Bacillus thuringiensis* in the insect model host *Galleria mellonella* results in epigenetic modifications. Virulence. (2017) 8:1618–30. doi: 10.1080/21505594.2017.1325975 PMC5810480 28521626

[25] Cime-Castillo J Arts RJW Vargas-Ponce de León V Moreno-Torres R Hernández-Martínez S Recio-Totoro B. DNA Synthesis is activated in mosquitoes and human monocytes during the induction of innate immune memory. Front Immunol. (2018) 9:2834. doi: 10.3389/fimmu.2018.02834 30555493 PMC6284063

[26] Quintin J Saeed S Martens JHA Giamarellos-Bourboulis EJ Ifrim DC Logie C. *Candida albicans* infection affords protection against reinfection *via* functional reprogramming of monocytes. Cell Host Microbe. (2012) 12:223–32. doi: 10.1016/j.chom.2012.06.006 PMC3864037 22901542

